# Human neural stem cell-derived neuron/astrocyte co-cultures respond to La Crosse virus infection with proinflammatory cytokines and chemokines

**DOI:** 10.1186/s12974-018-1356-5

**Published:** 2018-11-15

**Authors:** Brian E. Dawes, Junling Gao, Colm Atkins, Jacob T. Nelson, Kendra Johnson, Ping Wu, Alexander N. Freiberg

**Affiliations:** 1 Department of Microbiology and Immunology, University of Texas Medical Branch, Galveston, USA; 2 Department of Neuroscience, Cell Biology and Anatomy, University of Texas Medical Branch, Galveston, USA; 3 Department of Pathology, University of Texas Medical Branch, 301 University Boulevard, Galveston, 77555-0609 USA; 4 Center for Biodefense and Emerging Infectious Diseases, University of Texas Medical Branch, Galveston, USA; 5 Sealy Institute for Vaccine Sciences, University of Texas Medical Branch, Galveston, USA

**Keywords:** La Crosse virus, Neural stem cells, Neurons, Astrocytes, Inflammation, Encephalitis, Central nervous system

## Abstract

**Background:**

La Crosse virus (LACV) causes pediatric encephalitis in the USA. LACV induces severe inflammation in the central nervous system, but the recruitment of inflammatory cells is poorly understood. A deeper understanding of LACV-induced neural pathology is needed in order to develop treatment options. However, there is a severe limitation of relevant human neuronal cell models of LACV infection.

**Methods:**

We utilized human neural stem cell (hNSC)-derived neuron/astrocyte co-cultures to study LACV infection in disease-relevant primary cells. hNSCs were differentiated into neurons and astrocytes and infected with LACV. To characterize susceptibility and responses to infection, we measured viral titers and levels of viral RNA, performed immunofluorescence analysis to determine the cell types infected, performed apoptosis and cytotoxicity assays, and evaluated cellular responses to infection using qRT-PCR and Bioplex assays.

**Results:**

hNSC-derived neuron/astrocyte co-cultures were susceptible to LACV infection and displayed apoptotic responses as reported in previous in vitro and in vivo studies. Neurons and astrocytes are both targets of LACV infection, with neurons becoming the predominant target later in infection possibly due to astrocytic responses to IFN. Additionally, neuron/astrocyte co-cultures responded to LACV infection with strong proinflammatory cytokine, chemokine, as well as MMP-2, MMP-7, and TIMP-1 responses.

**Conclusions:**

hNSC-derived neuron/astrocyte co-cultures reproduce key aspects of LACV infection in humans and mice and are useful models to study encephalitic viruses. Specifically, we show astrocytes to be susceptible to LACV infection and that neurons and astrocytes are important drivers of the inflammatory responses seen in LACV infection through the production of proinflammatory cytokines and chemokines.

**Electronic supplementary material:**

The online version of this article (10.1186/s12974-018-1356-5) contains supplementary material, which is available to authorized users.

## Background

La Crosse virus (LACV), family *Peribunyaviridae* (genus *Orthobunyavirus*), is a leading cause of pediatric arboviral encephalitis in the USA [1]. The primary vector of LACV is the eastern tree-hole mosquito (*Ochlerotatus triseriatus*). LACV was responsible for 665 confirmed cases of encephalitis from 2003 to 2012, although the true incidence of disease is thought to be underestimated [2]. Endemic areas of infection include the Midwest and Appalachian regions, with county-level incidence of 0.2–228 cases per 100,000 children under the age of 15, but LACV is also becoming an important emerging pathogen of the Southern and Western United States [3]. Despite the threats posed, there are currently no approved therapeutics or vaccines available against LACV.

LACV encephalitis is almost exclusively found in children under 15 years of age [4]. Like other arboviruses, the majority of cases present as mild febrile illness, but in a minority of cases, LACV causes severe neuroinvasive disease including encephalitis, meningitis, and meningoencephalitis [5]. Neuroinvasive LACV typically presents with fever, headache, lethargy, and vomiting, and nearly half of patients experience seizures [4, 5]. While the disease is rarely (< 1%) fatal, neurological deficits such as epilepsy (in 10–28% of cases), reduced IQ, and attention-deficit-hyperactivity disorder (ADHD) are not uncommon [4–6].

LACV replicates peripherally and likely invades the central nervous system (CNS) via the olfactory bulb in the mouse model of LACV encephalitis after the compromise of the blood-brain barrier (BBB) [7]. In human infection, cortical and basal ganglia neurons appear to be the primary target of infection in the CNS leading to foci of neuronal necrosis [8]. Additionally, inflammatory lesions with largely monocytic infiltration and lymphocytic perivascular cuffing are noted [8]. The understanding of LACV neuropathogenesis has been advanced by studies using the suckling mouse model which closely resembles human disease including age-related susceptibility [9, 10]. Infection of adult mice and rhesus macaques result in asymptomatic infections and antibody responses [9, 10]. Most studies agree that neurons comprise the main target cell in the CNS [9, 11]. Infected neurons appear to undergo apoptosis via mitochondrial antiviral-signaling protein (MAVS)-induced oxidative stress [12]. However, some groups report low levels of astrocyte infection in vitro and in vivo [1, 11]. Especially interesting is the finding that when NSs, a LACV encoded interferon (IFN) antagonist, is deleted, astrocytes significantly increase production of IFN, suggesting that IFN production in astrocytes is antagonized by LACV [11]. Regarding the inflammatory component of the disease, a recent study showed that lymphocytes play a protective role during LACV infection of adult mice and do not contribute to the pathogenesis of weanling mice [13]. The majority of inflammatory cells noted in human and mouse brains during LACV infection are monocytes and macrophages. Recent work has demonstrated that in the mouse model, CCL2 is important for inflammatory monocytic migration within the brain and that astrocytes are a source of CCL2 in the brain [8, 14]. Importantly, it is becoming increasingly clear that CNS parenchymal cells play a major role in the development of innate immune responses during LACV infection [15–17]. Additionally, cytokine responses can also negatively impact BBB integrity and lead to worsened neuroinvasion [18, 19]. While our knowledge on the pathogenesis and molecular mechanisms of LACV-induced disease using animal models is increasing, there is still a need to verify many of these results with a human-based system.

Primary human neurons are terminally differentiated, post-mitotic, and difficult to obtain. Most studies of encephalitic viruses rely on primary rat or mouse neuronal cells or human neuroblastoma cell lines. While these models are strong tools for understanding pathogenesis, species differences and the genetic and signaling abnormalities found in these models require validation using human cells without genetic modification. Furthermore, most studies rely on the use of a single cell type, although it has been shown that neuronal cells behave differently in co-culture compared to monoculture [20, 21]. In recent years, human neural stem cells (hNSC), embryonic stem cells (hESCs), and induced pluripotent cells (iPCs) have become important tools in studying neurologic diseases, including encephalitic viruses. Varicella zoster virus (VZV) has been extensively studied using such systems, which has provided accurate models for VZV productive infection, latency, and reactivation. [22–26].

In this study, we use a well-validated hNSC-derived neuron/astrocyte co-culture system which has previously been used in the study of neurodegenerative diseases [27–29]. Importantly, this primary human neural cell system was recently used to assess Zika virus-induced changes in hNSC differentiation, although this study mainly focused on the direct infection of hNSCs rather than differentiated neuronal cells [30]. We have reported susceptibility of neuron/astrocyte co-cultures to infection with henipaviruses, but an in-depth characterization of the cellular responses to infection has not been reported yet [31]. In the present study, we infected hNSC-derived neuron/astrocyte co-cultures with LACV. Our results indicate that both neurons and astrocytes are highly susceptible to LACV, and that LACV infection induces strong proinflammatory responses, which likely play a major role in the observed neuroinflammation and breakdown in the BBB.

## Methods

### Cells and viruses

Vero CCL81 cells were acquired from American Type Culture Collection (ATCC, Manassas, VA). Vero cells were propagated using MEM (Corning) supplemented with 10%FBS. K048 hNSCs were originally obtained from the cortex of a 9-week-old male fetus and were propagated as described previously [27]. Briefly, hNSCs were cultured as nonadherent neurospheres in DMEM/F12 (Corning) media supplemented with epidermal growth factor (EGF) (20 ng/mL) (R&D Systems), fibroblast growth factor (FGF) (20 ng/mL) (R&D Systems), leukocyte inhibitory factor (LIF) (10 ng/mL) (Chemicon), heparin (5 μg/mL) (Sigma-Aldrich), and insulin (25 μg/mL) (Sigma-Aldrich). Cells were passaged every 10 days and maintained at 37 °C and 8.5% CO_2_.

hNSCs were plated onto wells coated with 0.01% poly-D-Lysine (Sigma-Aldrich) and 1 μg/cm^2^ laminin. Cells were primed for 4 days with a priming media containing EGF (20 ng/mL), LIF (10 ng/mL), and laminin (1 μg/mL) (GIBCO). K048 hNSCs were primed and differentiated into neurons and astrocytes in a roughly 1:1 ratio. The neurons in this system have previously been characterized as being both GABAergic and glutamatergic, and the overall composition is similar to that found in the cerebral cortex [32]. Cells were then differentiated for 9 days in a differentiation media containing N2 basal media supplemented with glutathione (1 μg/mL) (Sigma-Aldrich), biotin (0.1 μg/mL) (Sigma-Aldrich), superoxide dismutase (2.5 μg/mL) (Sigma-Aldrich), DL-α-tocopherol (1 μg/mL) (Sigma-Aldrich), DL-α-tocopherol acetate (1 μg/mL) (Sigma-Aldrich), and catalase (2.5 μg/mL) (Sigma-Aldrich).

LACV was obtained from the World Reference Center for Emerging Viruses and Arboviruses at the University of Texas Medical Branch (kindly provided by R. Tesh). The strain used was isolated from a human brain in Wisconsin in 1964. This strain had undergone nine passages in suckling mice and was amplified in our lab in one passage in Vero cells.

### Growth curves

Neuron/astrocyte co-cultures were infected with 0.1, 1, or 10 multiplicity of infection (MOI) of LACV for 1 h at 37 °C and 8.5% CO_2_. Virus inoculum was removed, cells gently washed with PBS, and fresh culture medium re-added. Cells only underwent one PBS wash due to the fragility of the neurons to avoid their detachment. Supernatant aliquots were then collected at various time points after infection. Samples were titrated via standard plaque assay. Cell culture supernatant aliquots were serially diluted in MEM supplemented with 2% FBS and used to infect Vero CCL81 cells for 1 h. Cells were washed with PBS and given a media overlay of MEM with 0.8% tragacanth (Sigma-Aldrich). At 4 days post-infection, the overlay was removed, cells were fixed in 10% formalin (Thermo Fisher) and stained using crystal violet (Thermo Fisher), and plaques were counted.

### Immunofluorescence

Neuron/astrocyte co-cultures on glass coverslips were infected with 0.1, 1, or 10 MOI of LACV as described above. Infected cells were fixed in formalin at various times post-infection. Cells were stained with primary antibodies and fluorophore-conjugated secondary antibodies along with DAPI (Sigma Aldrich) as previously described [20]. Antibodies used were rabbit-anti-microtubule associated protein 2 (MAP2) polyclonal (Millipore, AB5622) used at 1:500, rabbit-anti-glial fibrillary acidic protein (GFAP) polyclonal (Millipore, AB5804) used at 1:2,000, and mouse-anti-LACV Gc monoclonal (ThermoFisher, MA1–10801) used at 1:1,000. Secondary antibodies were Alexa Fluor goat-anti-rabbit 594 and goat-anti-mouse 488 used at 1:500 (ThermoFisher). Images were acquired on an Olympus IX71 fluorescent microscope and cells were quantified visually using six random fields per condition with an average of over 200 cells/field.

### Cytotoxicity and apoptosis assays

Neuron/astrocyte co-cultures were grown in 96-well plates and infected with 1 MOI of LACV as in other experiments along with media only controls and 10 μM staurosporine (Abcam) treatment. Cells were then assayed using the ApoTox-Glo triplex assay (Promega) according to the manufacturer’s protocols. Plates were assayed on a BioTek Synergy HT plate reader at 485nm_Ex_/505nm_Em_ for cytotoxicity (extracellular protease activity) and luminescence for apoptosis (caspase 3/7 activity).

### TUNEL assay

Neuron/astrocyte co-cultures were infected with 1 MOI LACV as described previously and fixed at 96 HPI. Additionally, staurosporine-treated cells were fixed at 12 h post-treatment. Formalin-fixed monolayers were prepared for immunofluorescence staining as previously described. Terminal deoxynucleotidyl transferase dUTP nick end labeling (TUNEL) staining (TACS 2 TdT-Fluor In Situ Apoptosis Detection Kit, R&D systems) was performed as per manufacturer instructions. Briefly, following DAPI stain, cells were incubated in labeling buffer, followed by labeling reaction mix. Strep-FITC was added for 20 min before final washes. Slides were briefly rinsed in ddH_2_O to remove residual salt and inverted onto Fluoromount G (Southern Biotech) and allowed to cure overnight at 4 °C in the dark. Samples were imaged on an Olympus BX61 microscope.

### BioPlex assays

Co-cultures were infected as in previous experiments with 0.1, 1, or 10 MOI of LACV or treated with heat-inactivated LACV (60 °C for 30 min) (1 MOI) or 10 μM polyinosinic:polycytidylic acid (poly I:C) (Sigma Aldrich). Supernatant samples were collected at various time points post-infection and γ-irradiated with a dose of 5 Mrad to inactivate the infectious virus. Cells treated with Poly I:C or heat-inactivated virus were collected at 48 h post-infection (HPI) only. Samples were then used for BioPlex assay analysis (Bio-Plex Pro Human Cytokine, Group 1, 27-Plex, Bio-Plex) according to manufacturer’s protocols. Standard curves were developed using fresh standards provided in each kit. The assays were run on a Bio-Plex 200 system (Bio-Rad), and data was analyzed using Bio-Plex Manager (Bio-Rad).

### qRT-PCR

Co-cultures were grown and infected with 0.1, 1, 10 MOI or treated with heat-inactivated virus or poly I:C. Cells were lysed and collected in TRIzol (Thermo Fisher) reagent, and RNA was isolated using Direct-zol RNA Miniprep kits (Zymo Research). cDNA was obtained using High Capacity RNA-to-cDNA kit (ThermoFisher). cDNA was then amplified using SYBR green mix (Bio-Rad) on a Bio-Rad CFX384 instrument. Data was analyzed using CFX Manager (Bio-Rad), and mRNA expression differences were determined via change in threshold cycle (ΔCT) and normalized to 18S RNA (ΔΔCT). PrimeTime qPCR primers (IDT) were used to target MMP7, MMP2, and TIMP2. PrimePCR PCR primers (Bio-Rad) were used to target IL-6, IL-8, CXCL10, CCL2, CCL4, CCL5, and TNF-α. Additional primers (IDT) were used to target 18S (For-GTAACCCGTTGAACCCCATT, Rev-CCATCCAATCGGTAGTAGCG), IFN-α (For-GACTCCATCTTGGCTGTGA, Rev-TGATTTCTGCTCTGACAACCT), IFN-β (For-TCTGGCACAGGTAGTAGGC, Rev-GAGAAGCACAACAGGAGAGCAA) and LACV (For-ATTCTACCCGCTGACCATTG, Rev-GTGAGAGTGCCATAGCGTTG).

### MMP activity assay

Supernatants of LACV-infected neuron/astrocyte co-cultures were assayed for the activities of matrix metalloproteinases (MMPs) using MMP Activity Assay Kit (Fluorometric-Green) (Abcam, #ab112146) as per manufacturer’s instructions. Briefly, pro-MMPs in solution are activated by incubating with APMA, immediately prior to enzyme reaction. Samples were read on a Bio-Tek Synergy multimode plate reader at 490/525 nm at 60 min incubation. Substrate and culture media controls were used to reduce background in analyzed values. All conditions were performed in singlicate from biological triplet samples.

### Statistical analysis

All experiments were performed in biological triplicate. All statistical analysis and figure preparation was performed with Prism (GraphPad Software). Cytotoxicity and apoptosis assays were subjected to two-way ANOVA with Bonferroni’s multiple comparisons test. BioPlex control experiments were subjected to one-way ANOVA with Tukey’s multiple comparisons test. The qRT-PCR and BioPlex experiments of the stimulated controls were subjected to one-way ANOVA with Dunnett’s multiple comparisons test. The qRT-PCR, BioPlex, MMP activity assays, and immunofluorescence experiments of virus-infected samples were subjected to two-way ANOVA with Tukey’s multiple comparisons test. TUNEL assays were subjected to one-way ANOVA with Tukey’s (percent of cells TUNEL positive) or Sidak’s (percent of TUNEL positive cells expressing GFAP or MAP2) multiple comparisons tests.

## Results

### hNSC-derived neuron/astrocyte co-cultures are susceptible to LACV infection

**Fig. 1:**
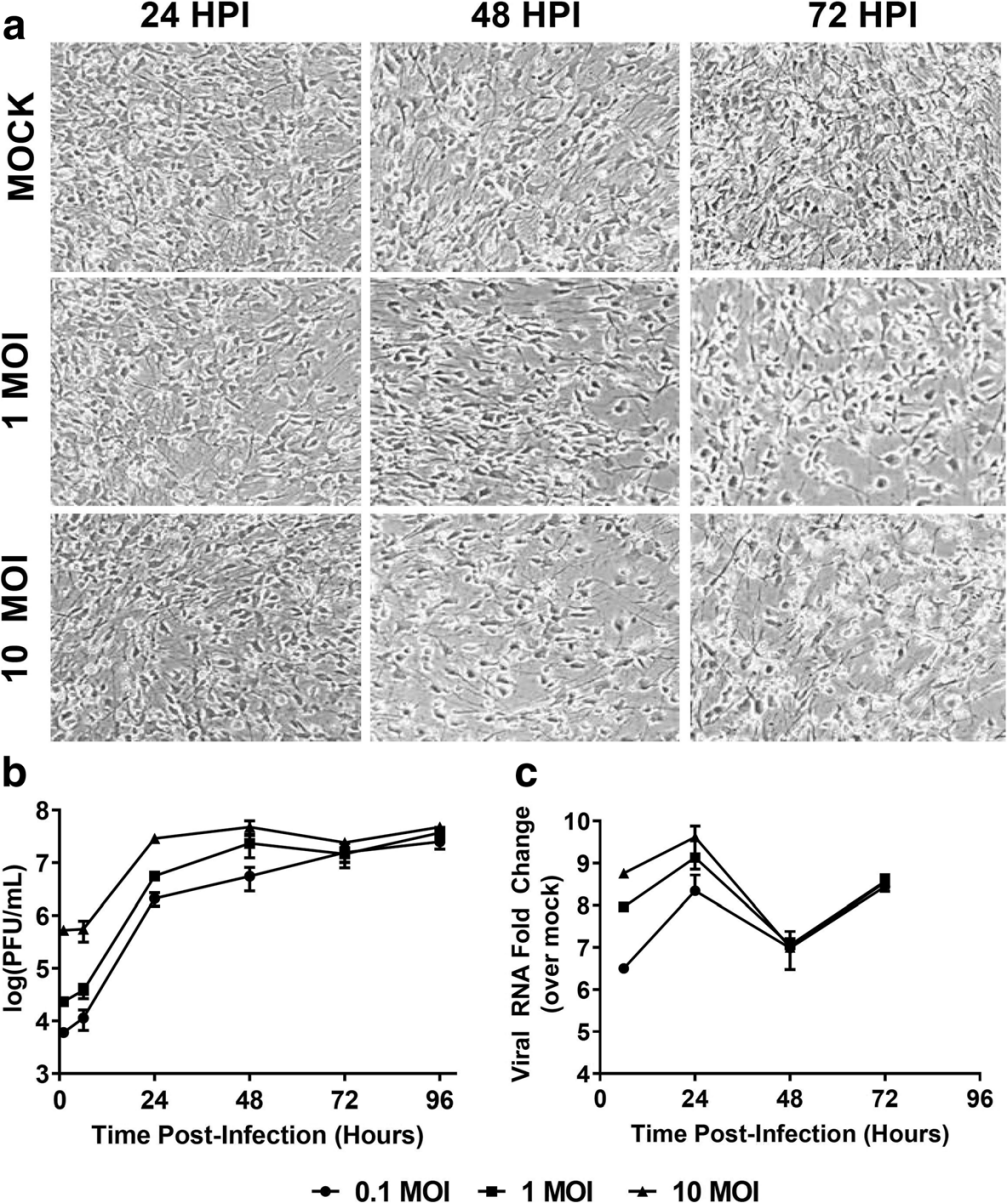
Susceptibility and replication kinetics in differentiated hNSC neuron/astrocyte co-cultures. hNSCs were primed and differentiated into mature neuron/astrocyte co-cultures for 9 days prior to infection with mock, 0.1, 1, or 10 MOI of LACV. **a** Co-cultures were imaged at various times post-infection via phase contrast microscopy. **b** Supernatant was collected at various times post-infection and titrated via plaque assay. **c** Cells were lysed and RNA was collected and qRT-PCR was used to determine viral replication. Anti-LACV N gene primers were used, and levels were determined as fold change relative to the mock-infected background

We first set out to demonstrate that hNSC-derived neuron/astrocyte co-cultures are susceptible to LACV infection and accurately replicate key aspects of LACV infection seen in other models including the susceptibility of neurons and apoptosis [9, 12, 33, 34]. Differentiated co-cultures were infected with 0.1, 1, or 10 MOI of LACV and images taken at 24, 48, and 72 HPI. At 48 HPI, the lytic nature of LACV infection could be observed, which is consistent with human and animal pathology (Fig. 1a) [8, 9, 33]. Cytopathic effect (CPE) was correlated with initial MOI, with 10 MOI infections resulting in maximum cell rounding and cell death as opposed to 0.1 MOI (not shown), which had only minimal observable CPE.

Supernatant aliquots were collected and titrated via plaque assay (Fig. 1b). High initial titers are due to the fragility of the co-culture system, and the limited ability to wash cells after the initial infection period. Regardless, a sharp increase in viral titers was observed within the first 24 HPI. The 10, 1, and 0.1 MOI infections reached peak titers by 24, 48, and 72 HPI, respectively. Peak titers were approximately 10^6^–10^7^ PFU/ml and remained within that range for the duration of the study. Additionally, we confirmed viral replication via qRT-PCR (Fig. 1c). An increase in viral RNA within the first 24 HPI was also noted; however, there was a decline at 48 HPI. By 72 HPI, viral RNA had once again increased. Our results indicate that the hNSC-derived neuron/astrocyte co-culture system is susceptible to LACV infection and supports replication and that viral infection leads to noticeable cytopathic effect.

### Neurons and astrocytes both support LACV replication

**Fig. 2:**
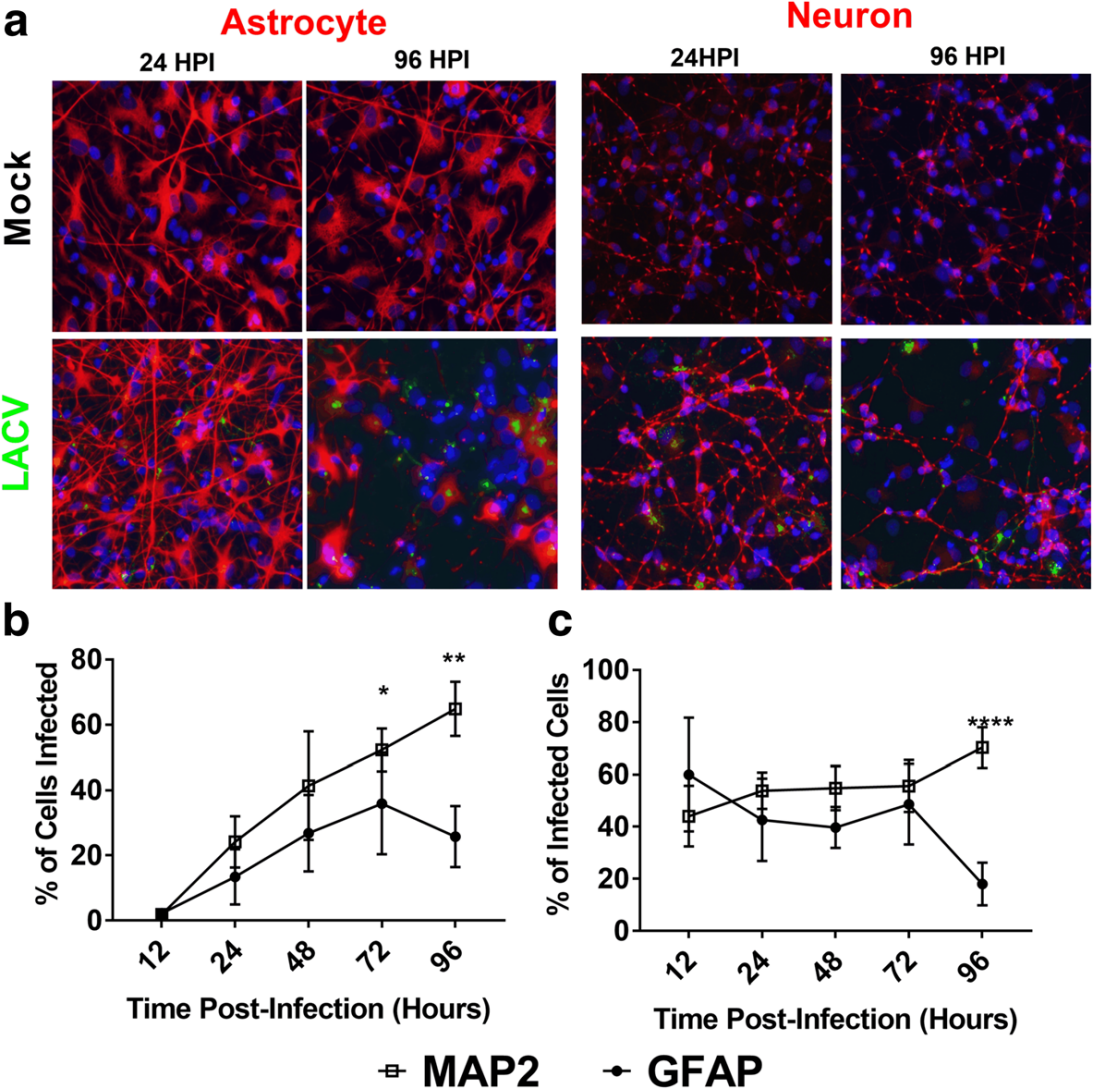
Identification of LACV target cells in neuron/astrocyte co-cultures. Neuron/astrocyte co-cultures were infected with 0.1, 1, and 10 MOI of LACV and formalin fixed at various times post-infection. **a** Cells were then stained for DAPI, LACV Gc protein, and either neuronal marker MAP2 or astrocytic marker GFAP. Representative images from the mock-infected and 1 MOI LACV-infected group are shown. **b** The percentage of neurons and astrocytes infected was determined over the course of infection for the 0.1 MOI group. The 1 and 10 MOI are available in Additional file 2: Figure S2a. **c** The percentage of infected cells expressing either MAP2 or GFAP was quantified for the set of cells infected with 0.1 MOI. The 1 and 10 MOI have similar trends and are available in Additional file 2: Figure S2b. *P < 0.05, **P < 0.01, ****P < 0.0001

We next determined the cell types infected with LACV in the neuron/astrocyte co-cultures. We stained infected cells against LACV glycoprotein and either an astrocytic marker (GFAP) or a neuronal marker (MAP2) with a DAPI counterstain (Fig. 2a). These experiments demonstrated that both neurons and astrocytes were infected. Interestingly, different distribution patterns were noted, particularly at 96 HPI. Neurons either had small punctuate patterns or cell-wide distribution of viral antigen, while in astrocytes antigen was much more apparent in a larger cluster. To further quantify the cell types infected, fields with evident infection were counted and ratios of neuron to astrocyte (Additional file 1: Figure S1), the percentage of neurons or astrocytes infected (Fig. 2b), and the ratio of neurons to astrocytes infected (Fig. 2c) determined. The data indicate that, as previously reported, there is an approximate 1:1 ratio of neurons to astrocytes present in the co-cultures (Additional file 1: Figure S1) [27, 30]. As early as 12 HPI, the virus was detectable in both cell types, and the percentage of cells infected increases in a time and dose-dependent manner (Fig. 2b). By 96 HPI, about 60% of neurons are infected, and 30% of astrocytes are infected. At early time points, neurons and astrocytes appear to be infected at an approximate 1:1 ratio with a trend towards greater neuronal infection (Fig. 2c). However, by 72 HPI, neurons became the more prevalent infected cell type. By 96 HPI, the ratio of neurons to astrocytes infected was approximately 2:1. At higher MOIs, this trend was weaker, with approximately equal percentages of neurons and astrocytes infected (Additional file 2: Figure S2a), but neurons become the predominant cell type infected later in infection (Additional file 2: Figure S2b). Taken together, these data indicate that in the hNSC-derived neuron/astrocyte co-culture system neurons and astrocytes are both susceptible to LACV infection, but neuronal infection is enriched later during the course of infection.

### LACV induces apoptosis in hNSC-derived neuron/astrocyte co-cultures

**Fig. 3:**
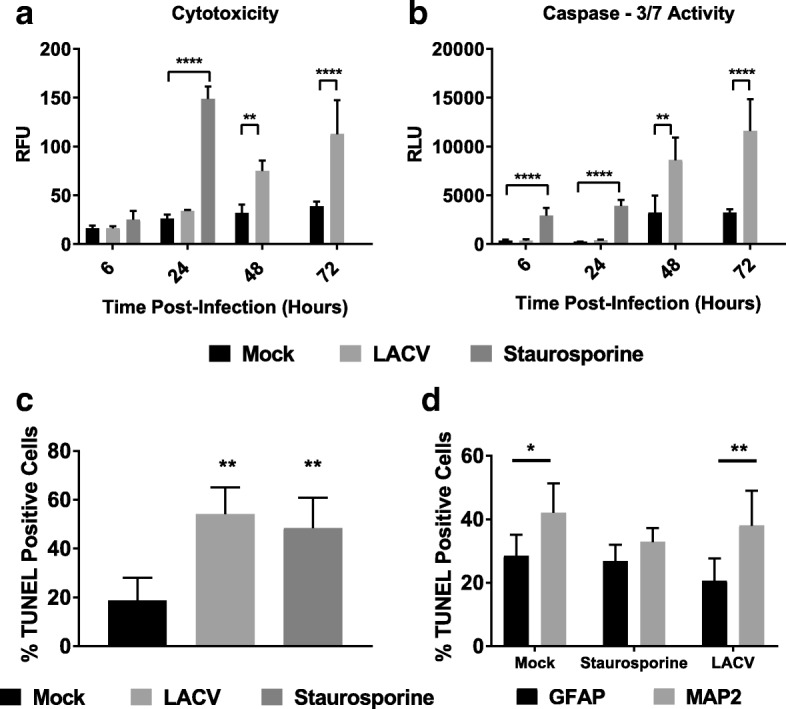
Apoptosis of LACV-infected neuron/astrocyte co-cultures. Neuron/astrocyte co-cultures were infected with 1 MOI of LACV and assayed for **a** cytotoxicity or **b** apoptosis at various time points. Staurosporine treatment was included up to 24 HPI as a positive control for apoptosis. Cytotoxicity was measured via fluorescence activity of a non-permeable protease substrate, and apoptosis was measured via luminescence from a caspase 3/7 substrate. **c** TUNEL staining was performed at 96 HPI to determine the percentage of apoptotic cells. **d** Double staining with TUNEL and cell markers was performed. Results reported are the percentages of TUNEL positive cells expressing either GFAP or MAP2. *P < 0.05 **P < 0.01 ****P < 0.0001

Previous studies have demonstrated that neurons in vivo and in vitro undergo apoptosis in response to LACV infection [9, 12, 33]. To further validate this system, we performed necrosis and apoptosis assays to confirm apoptotic cell death. Neuron/astrocyte co-cultures were either mock-infected, infected with LACV, or treated with staurosporine. A single dose (1 MOI) was selected because at this MOI CPE is apparent, titers are comparable to 10 MOI infections, and cells remain viable for longer which allowed us to assay later time points. A cytotoxicity assay (Fig. 3a) assessed supernatant for cell-impermeable proteases via fluorescence, and an apoptosis assay (Fig. 3b) detected caspase 3/7 activity via luminescence. As expected, mock-infected controls displayed low levels of cytotoxicity and caspase activation throughout the course of the experiment. Staurosporine controls were only measured for the first 24 h but demonstrated significant cytotoxicity and caspase 3/7 activation at that time point indicating an apoptotic response. LACV-infected co-cultures began displaying significant cytotoxicity at 48 HPI (Fig. 3a) consistent with CPE observed in Fig. 1a. Additionally, at 48 HPI, significant increases in caspase 3/7 activity were measured in LACV-infected neuron/astrocyte co-cultures (Fig. 3b). Taken together, these results indicate that apoptosis is a primary mediator of cytotoxicity in LACV-infected neuron/astrocyte co-cultures.

To determine which cells were dying as a result of apoptosis, we performed TUNEL staining on LACV-infected neuron/astrocyte co-cultures at 96 HPI. Around 20% of cells at 96 HPI were apoptotic in the absence of LACV infection, and staurosporine treatment led to roughly 50% of cells undergoing apoptosis (Fig. 3c). LACV infection resulted in similar levels of apoptosis to staurosporine treatment (Fig. 3c). To determine which cells were undergoing apoptosis, cells were double stained with TUNEL staining and either GFAP or MAP2 (Fig. 3d). Apoptotic cells in mock-infected co-cultures were both MAP2 and GFAP positive, but neurons appeared to be more susceptible. Staurosporine induced apoptosis in both neurons and astrocytes equally. LACV induced apoptosis in both neurons and astrocytes. However, apoptotic cells were nearly twice as likely to be neurons. These data indicate that LACV infection induces apoptosis in both neurons and astrocytes, but neurons appear to be more susceptible to apoptosis.

### hNSC-derived neuron/astrocyte co-cultures are responsive to proinflammatory stimuli

**Fig. 4:**
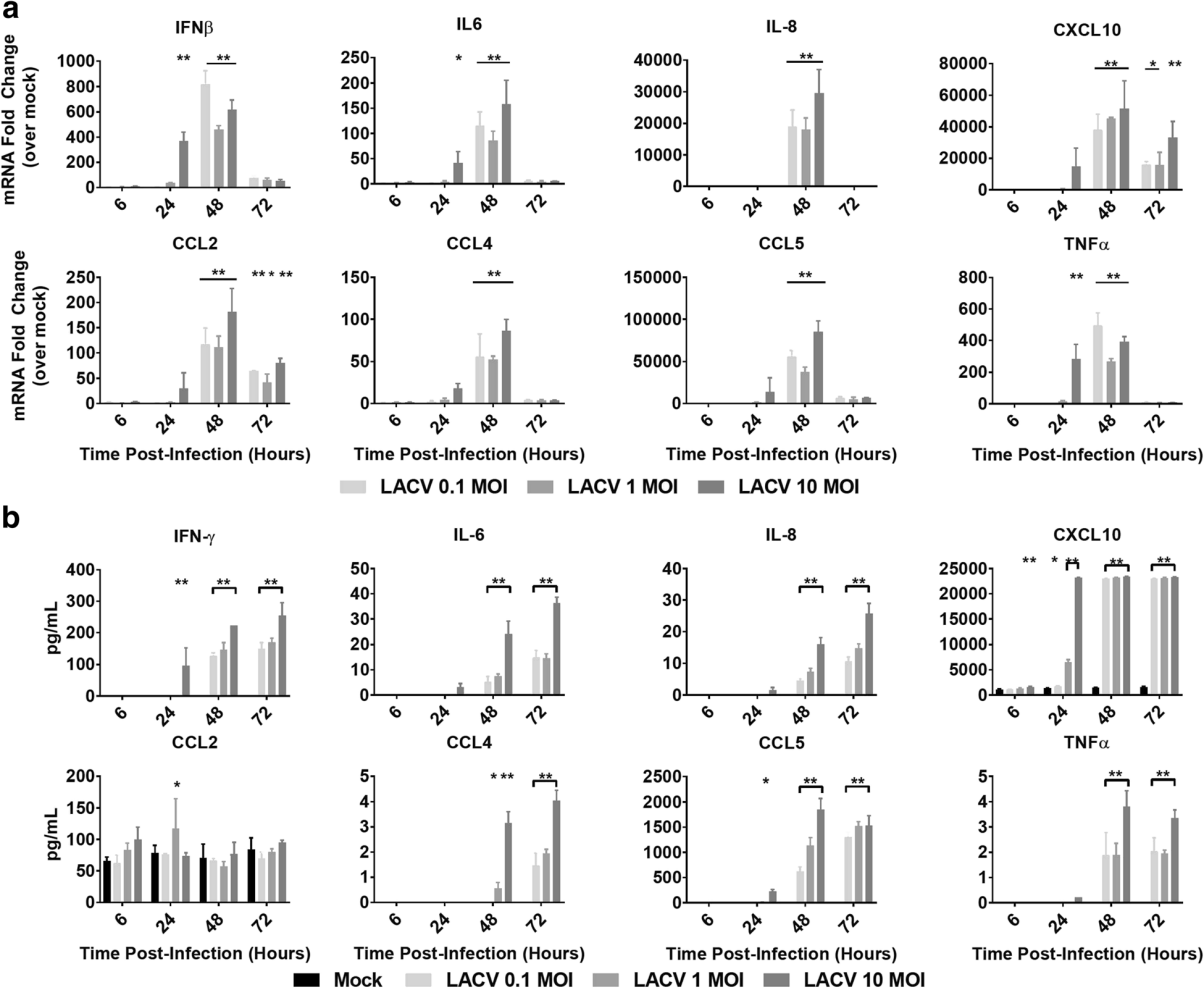
Cytokine and chemokine responses of neuron/astrocyte co-cultures to LACV infection. Cells were either infected with 0.1, 1, or 10 MOI of LACV. **a** Cells were lysed and RNA was collected and assessed for changes in selected cytokine/chemokine expression via qRT-PCR. Values are reported as fold change relative to mock treatment normalized to 18S RNA. **b** Supernatant was collected and assayed for changes in selected cytokine/chemokine secretion via BioPlex assay *P < 0.5, **P < 0.01

After demonstrating that the hNSC-derived neuron/astrocyte co-culture system reproduced the basic pathology seen in LACV, we next confirmed the innate immune responses of these primary human cells to inflammatory stimuli [8, 9, 33]. For these studies, neuron/astrocyte co-cultures were either mock-treated or treated with heat-inactivated LACV or Poly I:C (Additional file 3: Figure S3). No significant increases were noted with inactivated LACV for any of the tested analytes at the transcriptional level (Additional file 3: Figure S3a). In contrast, the majority of analytes were significantly upregulated after Poly I:C exposure including IFN-β, proinflammatory cytokines IL-6 and TNF-α, and proinflammatory chemokines IL-8, CCL2, CCL4, CCL5, and CXCL10. IFN-α was not significantly upregulated in response to Poly I:C similar to previous studies [35, 36]. Translational changes were confirmed using BioPlex assays (Additional file 3: Figure S3b). Among the upregulated proteins were the proinflammatory cytokines IFN-γ, IL-6, and TNF-α and the proinflammatory chemokines IL-8, CCL4, CCL5, and CXCL10 (Fig. 4b). Surprisingly, no significant increase in CCL2 protein expression was detected in comparison to mock- and heat-inactivated LACV-treated cells. These data indicate that neuron/astrocyte co-cultures have the ability to respond to proinflammatory stimuli in a physiologically relevant manner with the production of interferons, cytokines, and chemokines.

### hNSC-derived neuron/astrocyte co-cultures respond to LACV infection with proinflammatory chemokine and cytokine responses

After demonstrating that hNSC derived neuron/astrocyte co-cultures are capable of responding to insult with the production of cytokines and chemokines, we measured the co-cultures’ responses to LACV infection. To accomplish this, co-cultures were infected with 0.1, 1, or 10 MOI of LACV and responses measured over time. RNA was collected and analyzed via qRT-PCR. IFN-α responses were detected as early as 6 HPI and continued until 48 HPI before decreasing to near baseline levels at 72 HPI (Additional file 4: Figure S4a). IFN-β responses were detected later (24 HPI through 48 HPI) but were much stronger (Fig. 4a). The other cytokines and chemokines investigated (IL-6, TNF-α, IL-8, CCL2, CCL4, CCL5, and CXCL10) displayed significant increases with the peak mRNA induction at 48 HPI (Fig. 4a). In general, these responses were stronger in the 10 MOI group than in the 0.1 and 1 MOI groups. Additionally, responses in the 10 MOI group had a tendency to show significant upregulation earlier (24 HPI), which correlated with the viral growth kinetics (Fig. 1b, c).

Protein secretion patterns closely mirrored mRNA responses. Most measured analytes had significant upregulation at 48 and 72 HPI, with some analytes such as IFN-γ, CXCL10, and CCL5 responding at 24 HPI (Fig. 4b). In general, the responses were stronger in the 10 MOI group. Interestingly, again, we noted no significant differences in CCL2 secretion opposed to large mRNA increases. PDGF, IL-1Rα, IL-4, IL-7, IL-9, Eotaxin, FGF, G-CSF, VEGF, MIP-1α, and GM-CSF also displayed upregulation at 48 and 72 HPI, IL-12, IL-10, and IL-13 displayed mild increases over mock, and IL-1β, IL-2, IL-5, IL-15, and IL-17 did not display significant increases (Additional file 4: Figure S4b). These data demonstrate that neuron/astrocyte co-culture responded to LACV infection with a strong pro-inflammatory cytokine profile, with particularly large increases in monocytic and lymphocytic chemokines.

### hNSC-derived neuron/astrocyte co-cultures alter MMP and TIMP expression in response to LACV infection

**Fig. 5:**
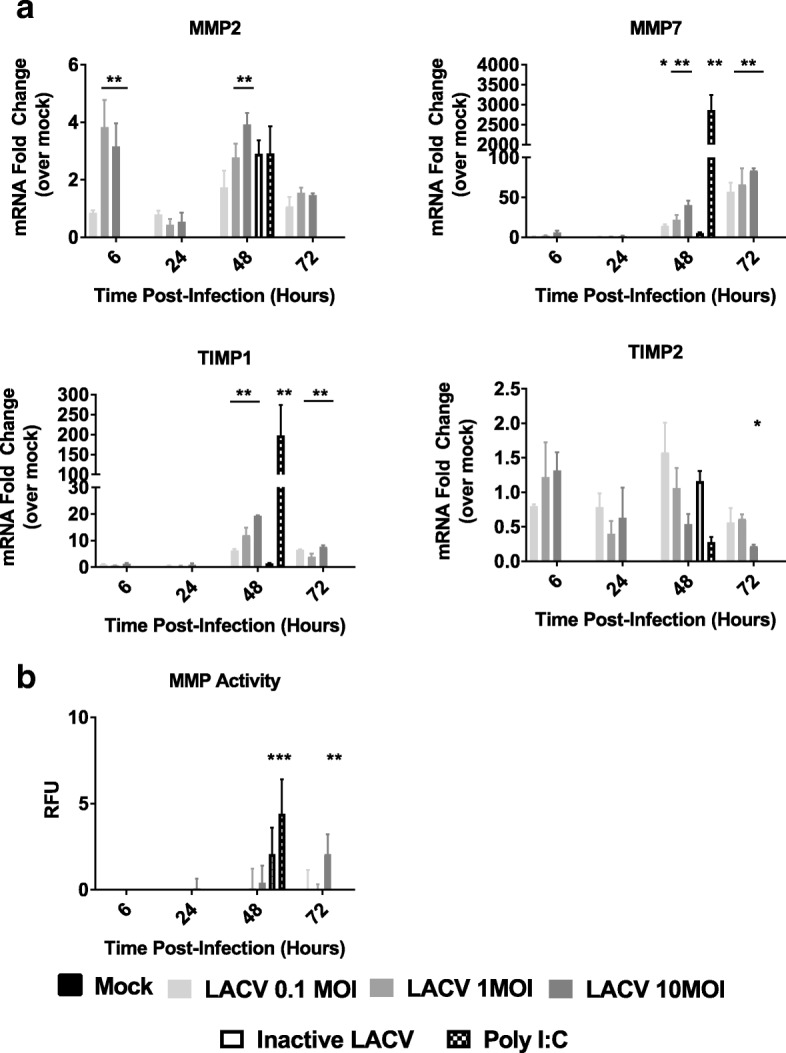
MMP and TIMP responses of LACV infected neuron/astrocyte co-cultures. Neuron/astrocyte co-cultures were either mock infected, treated with inactivated LACV, treated with Poly I:C, or infected with 0.1, 1, 10 MOI of LACV. **a** RNA was collected and analyzed via qRT-PCR against selected MMPs and TIMPs. Values are reported as fold change relative to mock treatment normalized to 18S RNA. **b** MMP activity assays were performed to determine MMP cleavage activity. *P < 0.5, **P < 0.01

In addition to cytokine and chemokine responses, we also determined expression levels of matrix metalloproteinases (MMPs) or tissue inhibitor of metalloproteinases (TIMPs), which can influence the permeability of the BBB [37–39]. To determine the changes in MMP and TIMP expression in the neuron/astrocyte co-culture system, cells were treated as in previous experiments and RNA analyzed by qRT-PCR. Here, we assayed MMP2, MMP7, MMP9, TIMP1, and TIMP2, which have previously been described to be differentially expressed after infection of the CNS [19, 40–42]. No responses were measured for MMP9 (data not shown). Low but significant increases were detected for MMP2, although interestingly, Poly I:C failed to induce a significant change (Fig. 5a). Of note, the increase at 48 HPI, while statistically significant, was very similar to that of inactive virus controls, and may constitute a measurement artifact. MMP7 had the largest induction with significant upregulation for all three MOIs at 48 and 72 HPI (Fig. 5a). TIMP-1 was also significantly upregulated in response to viral infection and Poly I:C at 48 and 72 HPI. Lastly, upregulation was not noted for TIMP2. We instead measured significant downregulation of TIMP2 mRNA for co-cultures infected with 10 MOI at 72 HPI (Fig. 5a). Additionally, while not significant, Poly I:C treatment trended to similar levels of downregulation. Taken together, these data suggest that later responses in neurons and/or astrocytes significantly upregulate MMP7 and TIMP1 while potentially downregulating TIMP2.

As both MMPs and TIMPs were upregulated, we assessed the overall MMP activity during LACV infection via a MMP activity assay. Supernatant from LACV infected, inactivated LACV infected, or Poly I:C treated co-cultures was assessed for MMP substrate cleavage activity throughout the course of infection (Fig. 5b). Poly I:C and inactive virus was only assayed at 48 HPI. Poly I:C and inactive virus significantly increased MMP substrate cleavage activity at 48 HPI. LACV failed to induce any changes in MMP activity until 72 HPI, and only at the highest MOI. These results suggest that while LACV does increase MMP expression, these changes do not result in a functional change until very late during the course of infection.

## Discussion

CNS infections continue to be an important facet of emerging viral disease. Despite this threat, in vitro models of CNS infection remain limited. Clinical samples are rarely obtained outside of autopsy due to the challenging nature of CNS biopsy. Animal models, both in vivo and ex vivo studies with primary cells, have been the primary models and provided many important insights [43, 44]. However, animal models often do not accurately model all aspects of clinical disease described in patients. Many studies have now demonstrated profound differences between murine and human cellular responses [45, 46]. Differences also exist in the structure and function of human and murine parenchymal cells. For example, astrocytes are much larger and perform more complex signaling in humans than in mice [47]. Human primary CNS cells are relevant models, but they are difficult to obtain and introduce donor variability. Immortalized cell lines offer a useful alternative for in vitro studies, but the interpretation of results is limited by alterations in specific pathways and differences among different cell lines. Here, we report the use of a primary hNSC-derived neuron/astrocyte cell co-culture system as a more accurate and reproducible in vitro model to study encephalitic viruses. In addition to allowing for the repeated study of human primary cells without donor variability, co-culturing neurons and astrocytes allows for a more physiologically relevant model than neuronal monoculture. While the lack of donor variability is useful for the identification of pathogenic mechanisms, it may also obscure different aspects of disease as seen in other studies where different hNSC strains responded differently to infection [30]. Astrocytes play a key role in the physiologic support of neuronal health and function such as synaptic development, protection from glutamate toxicity and oxidative stress, and metabolic support [48]. Astrocytes also are increasingly recognized as major contributors to inflammatory responses during CNS infection. In addition to pathogenesis studies, neuron/astrocyte co-cultures may provide a relevant in vitro system for the evaluation of antiviral therapies against neurotropic viruses.

Neuron/astrocyte co-cultures were susceptible to LACV infection (Fig. 1) and high viral titers were reached regardless of initial viral inoculum (Fig. 1b). However, there was a drop in viral RNA measured at 48 HPI (Fig. 1c), for which the reasons are unknown, but the timing coincides with the peak IFN-β response suggesting that cells may become less susceptible to infection during this period (Fig. 4a). Future studies should evaluate the role of IFNs and the viral IFN antagonist NSs during LACV neuron/astrocyte infection. CPE was also observed at later time points consisting of cell rounding in detachment (Fig. 1a). Indeed, cytotoxicity and apoptosis assays indicate increased numbers of apoptotic cells beginning at 48 HPI (Fig. 3). Additionally, it seems that neurons are more susceptible to apoptosis than astrocytes in response to LACV infection (Fig. 3d). These data mirror those seen in previous work using murine models and the human NT2N cell line [9, 12, 33, 34]. Of note, while cytotoxicity and apoptosis were noted, large amounts of viable cells remain as late as 96 HPI. This contrasts reports of LACV as a highly cytopathic virus in mouse primary neurons and human NT2N cells and are likely due to intact IFN responses and the protective effects of astrocytic co-culture resulting in a more physiologic response and greater resilience to infection [12, 33].

The overall percentage of cells infected during this study was lower than expected, reaching maximums of 64% neuronal infection and 50% astrocytic infection (Fig. 2b). Again, this is likely due to intact antiviral sensing pathways and IFN responses. Multiple studies have indicated that both neurons and astrocytes are important producers of type I IFN during LACV infection, but astrocytes appear to be responsible for greater responses [11, 15]. Therefore, astrocytic IFN production may be responsible for limiting the magnitude of infection. The current study also demonstrates neurons as astrocytes are both highly susceptible to LACV infection. Neurons have long been recognized as a target of infection, but astrocytic infection has been largely ignored [8, 9, 11, 15]. One study demonstrated that in the weanling mouse model of LACV encephalitis, less than 1% of infected cells were astrocytes [11]. However, in the same study, deleting the LACV NSs gene (an IFN antagonist) resulted in large increases of astrocytic IFN-β production suggesting nonproductive infections or infections below the detection limit of utilized assays may be higher than previously thought [11]. Another group has proposed in a review that astrocytic infections are common and that the rapid death of infected astrocytes leads to a lack of detection in vivo [1]. Here, we have shown that human astrocytes are indeed highly susceptible to LACV infection in vitro (Fig. 2). At early time points, neurons and astrocytes are infected at a nearly 1:1 ratio, and at an MOI of 10, the infection in astrocytes is significantly higher than in neurons at 12 HPI (Additional file 2: Figure S2c). However, by 72 HPI, neurons became the predominant cell type infected in the 0.01 MOI infections. Despite the shifting tropism of viral infection, no changes were detected in the overall ratio of neurons to astrocytes, and astrocytes seemed to be less susceptible to apoptosis compared to neurons (Additional file 1: Figure S1, Fig. 3d). This suggests that rapid astrocytic death is not a major driver in the shift in tropism in vitro. This shift may be due to the type I IFN response which begins at 48 HPI. Astrocytes may be more sensitive to type I IFN signaling and induce strong antiviral states, protecting this cell type while leaving neurons more vulnerable. This shift is less prominent at higher MOIs, likely because too many cells are infected early to adequately demonstrate changes in tropism and/or IFN responses were mounted too late to counter the heavy infection. However, when comparing only infected cells, neurons still tend to dominate at later timepoints even at late MOIs (Additional file 2: Figure S2b). Future studies will attempt to determine the relative roles of IFN on neurons and astrocytes during viral infection.

Neurons and astrocytes are increasingly recognized as important components of CNS immune responses. We tested the responsiveness of hNSC-derived neuron/astrocyte co-cultures by stimulating with the inactivated virus and a dsRNA analogue, Poly I:C (Additional file 3: Figure S3). The co-cultures are highly responsive to Poly I:C, responding with large increases in IFNs and proinflammatory cytokines and chemokines at both the RNA and protein level. It is reasonable to conclude that these cells have intact antiviral sensing pathways and are capable of initiating inflammatory responses. No responses to inactivated LACV were detected, suggesting that viral replication is necessary for such cellular responses. Alternatively, these results may indicate that viral entry is necessary as heat inactivation may denature attachment and entry glycoproteins. Another interesting finding was that while CCL2 mRNA is induced following Poly I:C treatment, the levels of CCL2 secretion remain unchanged. This suggests additional levels of regulation of CCL2 at the translational level.

LACV infection generated similar, but more limited, proinflammatory responses in these neuron/astrocyte co-cultures with mRNA levels peaking around 48 HPI and protein levels closely mirroring (Fig. 4 and Additional file 4: Figure S4). Interestingly, LACV, but not Poly I:C induced an increase in IFN-α mRNA similar to previous studies [35, 36]. At the same time, unlike Poly I:C, LACV failed to induce IL-1β, IL-2, IL-5, IL-15, and IL-17. Additionally, LACV only resulted in modest increases in IL-10, IL-12, IL-9, and IL-13 only at late time points with high MOIs. This may be explained as either viral inhibition of select responses, lower levels of stimulation relative to Poly I:C, or differences in signaling pathways used to detect Poly I:C versus LACV. Indeed, Poly I:C was added to the supernatant without transfection, which typically stimulates TLR3 while LACV infection likely stimulates a larger range of endosomal and cytosolic RNA sensing receptors. These results clearly show that neurons and astrocytes are likely important factors in shaping the immune response to LACV encephalitis. This has been demonstrated for the type I IFN responses, but not yet the proinflammatory chemokines [11, 15].

Human pathology reports and animal models note inflammation primarily composed of macrophages and lymphocytes [8, 9, 14]. A recent study by Winkler et al. revealed that lymphocytes do not appear to be important in the development of neuropathology [13]. The same group has also shown inflammatory monocytes to be the primary infiltrating cell type, although their role in immune-mediated neuropathology was not addressed [14]. In the same study, it was noted that CCR2 was necessary for monocytes to migrate to lesions in the CNS, but not for recruitment to the CNS. Interestingly, while changes in mRNA expression for CCL2 were noted, protein expression in the cell culture supernatant was unchanged. Neurons and astrocytes constitutively express low levels of CCL2 in the healthy brain, as seen in our results, but some studies have noted a microglial requirement for upregulation after infection [17, 49]. Microglia are missing from this system and should be further evaluated for their effect on responses after LACV infection. Their response is potentially via bystander effects, as preliminary experiments infecting primary human microglia did not yield productive infection or chemokine responses (data not shown). Future studies should address the role of inflammatory monocytes during LACV encephalitis.

The strongest responses in our study were in the monocytic and lymphocytic chemokines, CCL5 and CXCL10. These chemokines have been associated with a wide range of viral CNS infections and are important for lymphocyte recruitment [50]. As previously mentioned, lymphocytes do not appear to play a role in immunopathology, but it is likely that during human infection their role is critical in the recovery from LACV encephalitis [13]. The receptors for these CCL5 and CXCL10 (CCR5 and CXCR3, respectively) have been shown to be critical for T cell recruitment for several encephalitic viruses such as West Nile virus (WNV), murine hepatitis virus (MHV), and herpes simplex virus (HSV) [51–53]. CCR5 has also been shown to be important for the control of WNV [54]. Our data therefore suggest that neurons and/or astrocytes are important for the recruitment of T cells to the CNS during LACV encephalitis, although in vivo studies will be required to confirm this hypothesis. Additionally, we show strong IFN-γ responses which have been shown to be critical for the control of several viral CNS infections such as HSV, Sindbis virus, measles virus, and Theiler’s murine encephalomyelitis virus [55–58].

One limitation of the current study is the difficulty in determining which cell types are responsible for signaling. Predictions can be made using other models, but as cells behave differently in co-culture, simple assessment of primary astrocytes and neurons may not be accurate. Both cell types appear to be important for cytokine responses in WNV infection, with astrocytes producing CXCL10, CCL2, and CCL5, but not IL-1β or IL-6 [59]. Neuronal cells were then shown to produce IL-1β, IL-6, IL-8, and TNF-α [60]. Both neurons and astrocytes appear to be important producers of CXCL10 during viral infection [59, 61]. However, these differences are often virus-specific, with H7N9 influenza inducing IL-6 and IL-8 in both neuronal and astrocytic cells [62]. Another layer of complexity is that host species differences are known to exist in cytokine expression and IFN responses [63]. These conflicting data highlight the need for species-specific models for viral infection. However, immunofluorescent or immunohistochemical staining for cytokines and chemokines is often problematic due to low intercellular levels of protein, and separation via FACS is difficult due to the fragility of the cells. Future effort needs to focus on better understanding cell-specific responses.

The neuron/astrocyte co-cultures in this system also demonstrated a potential to disrupt the BBB. While initial LACV neuroinvasion is thought to occur via hematogenous spread through capillaries in the olfactory bulb, further disruption of the BBB after neuroinvasion may contribute to greater viral neuroinvasion or increased inflammatory responses leading to greater damage [7]. Rift Valley fever virus, a related bunyavirus, is likely to also use the olfactory bulb for CNS entry, but generally maintains BBB integrity during infection in contrast to LACV [64, 65]. TNF-α, IL-6, and VEGF had modest upregulation in this study and have long been associated with increased BBB permeability (Fig. 5 and Additional file 4: Figure S4) [18, 19]. We additionally assessed MMPs and TIMPs (Fig. 5). MMP9 and MMP2 are typically the primary MMPs associated with BBB disruption [19]. Initial BioPlex screens and RT-PCR did not detect MMP9 after virus infection (data not shown). We did however observe modest increases in MMP2 at 6 HPI and large increases in MMP7. MMP7 has not been commonly studied in the context of viral encephalitis and its relevance is unknown. However, MMP7 is important for leukocyte infiltration during experimental autoimmune encephalomyelitis and is found in the CSF during AIDS dementia [40, 41]. TIMP-2 is constitutively expressed in the brain while TIMP-1 is inducible [42]. The current study demonstrates that TIMP-1 is induced following LACV infection and that TIMP-2 appears to be downregulated (Fig. 5a). As both MMPs and TIMPs are upregulated, the functional status of MMP activity required further study. An MMP substrate cleavage assay revealed that high MOI LACV infection could induce an increased MMP response at 96 HPI. However, this is very late in infection, and the MMP activity was not high (Fig. 5b). This suggests that TIMP upregulation may be limiting BBB breakdown by MMP upregulation during LACV infection, but the functional activity of these enzymes on the BBB during LACV infection in vivo remains to be assessed.

## Conclusion

Our results demonstrate the feasibility, accuracy, and usefulness of hNSC-derived neuron/astrocyte co-cultures to study encephalitic viruses. In the current study, many aspects of LACV encephalitis such as neurotropism and apoptosis were replicated. In addition, we noted the susceptibility of astrocytes early in infection with a shifting tropism later during the course of infection. This may explain why while low levels of astrocyte infection have been noted in experimental models, they do not appear to be prevalent in vivo. We also demonstrate intact viral sensing pathways and proinflammatory cytokine and chemokine responses. These neuron/astrocyte responses may drive the observed monocytic and lymphocytic infiltration observed during LACV encephalitis as well as prolonged disruption of the BBB.

## Additional files

Additional file 1: Figure S1. Neurons and astrocytes are present in a 1:1 ratio, which is not altered during LACV infection. Neuron/astrocyte co-cultures were either mock infected or infected with 0.1, 1, or 10 MOI of LACV. Cells were formalin fixed and stained for MAP2 or GFAP with DAPI counterstain. Percentages of GFAP or MAP2 positive cells were calculated across 6 fields of at least 200 cells. (PDF 187 kb)

Additional file 2: Figure S2. Neurons and astrocytes are both targets of LACV infection across various MOIs. Neuron/astrocyte co-cultures were either mock infected or infected with 0.1 or 10 MOI of LACV. Cells were formalin fixed and stained for MAP2 or GFAP, LACV antigen and with DAPI counterstain. (a) Percentages of neurons and astrocytes infected with LACV were calculated (b) Percentages of infected cells positive for GFAP and MAP2 were calculated. *P < 0.05, **P < 0.01 (PDF 38 kb)

Additional file 3: Figure S3. Innate immune responses to inflammatory stimuli in neuron/astrocyte co-cultures. RT-PCR and BioPlex assays were performed to determine the cytokine and chemokine responses of neuron/astrocyte co-cultures. (a) Co-cultures were treated with either mock, Poly I:C, or heat inactivated LACV and at 48 HPI assessed for changes in gene expression for selected cytokines/chemokines via qRT-PCR. Values are reported as fold change relative to mock treatment normalized to 18S RNA. (b) Supernatant was collected and assayed for changes in selected cytokine/chemokine secretion via BioPlex assay. * *P* < 0.5, **P < 0.01, ***P < 0.001, ****P < 0.0001. (PDF 207 kb)

Additional file 4: Figure S4. Full cytokine and chemokine responses of neuron/astrocyte co-cultures to LACV infection. Cells were either infected with 0.1, 1, or 10 MOI of LACV. (a) Cells were lysed and RNA was collected and assessed for changes in selected cytokine/chemokine expression via qRT-PCR. Values are reported as fold change relative to mock treatment normalized to 18S. (b) Supernatant was collected and assayed for changes in selected cytokine/chemokine secretion via BioPlex assay *P < 0.5, **P < 0.01. (PDF 461 kb)

## Abbreviations

ADHD: Attention-deficit-hyperactivity disorder
BBB: Blood-brain barrier
CNS: Central nervous system
CPE: Cytopathic effect
EGF: Epidermal growth factor
FGF: Fibroblast growth factor
GFAP: Glial fibrillary acidic protein
hESCs: Embryonic stem cells
hNSC: Human neural stem cells
HPI: Hours post-infection
HSV: Herpes simplex virus
IFN: Interferon
iPCs: Induced pluripotent cells
LACV: La Crosse virus
LIF: Leukocyte inhibitory factor
MAPS 2: Microtubule associated protein 2
MAVS: Mitochondrial antiviral-signaling protein
MHV: Murine hepatitis virus
MMPs: Metalloproteinases
MOI: Multiplicity of infection
PFU: Plaque forming units
Poly I:C: Polyinosinic:polycytidylic acid
TIMPs: Tissue inhibitor of metalloproteinases
TUNEL: Terminal deoxynucleotidyl transferase dUTP nick end labeling
VZV: Varicella zoster virus
WNV: West Nile virus
