# Supplementary figures and images for: Human neural stem cell-derived neuron/astrocyte co-cultures respond to La Crosse virus infection with proinflammatory cytokines and chemokines
Source: J Neuroinflammation. 2018 Nov 15;15:315. doi: 10.1186/s12974-018-1356-5 (PMC6236894; doi:10.1186/s12974-018-1356-5)

# Additional Figure 1

a.)

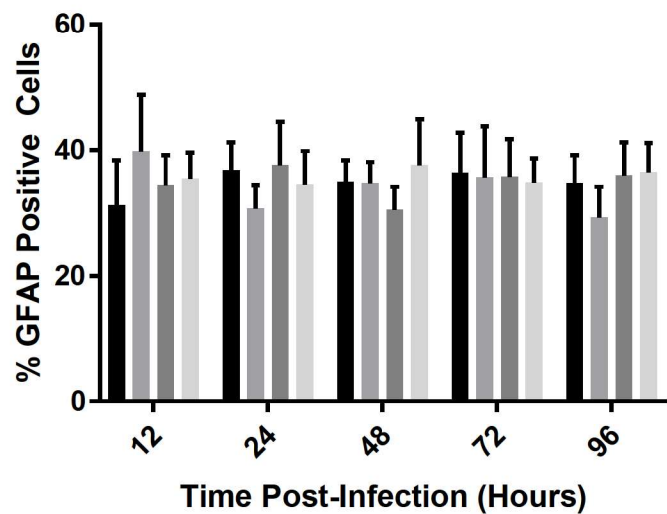

b.)

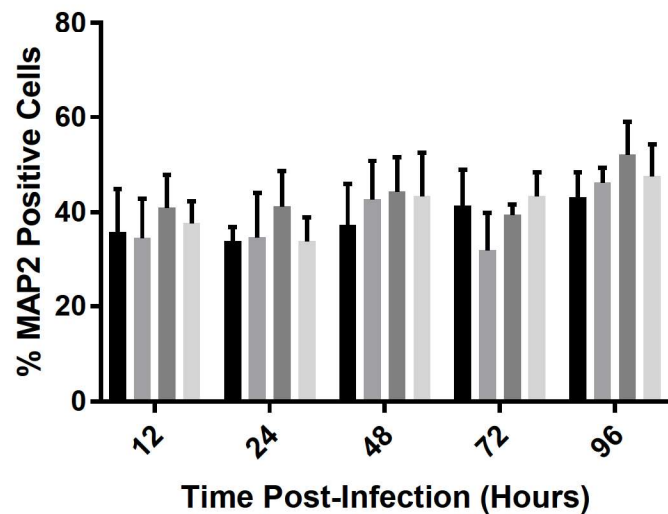

Mock 0.1 MOI 1 MOI 10 MOI

Supplement: Supplementary file 1 — Figure S1. Neurons and astrocytes are present in a 1:1 ratio, which is not altered during LACV infection. Neuron/astrocyte co-cultures were either mock infected or infected with 0.1, 1, or 10 MOI of LACV. Cells were formalin fixed and stained for MAP2 or GFAP with DAPI counterstain. Percentages of GFAP or MAP2 positive cells were calculated across 6 fields of at least 200 cells. (PDF 187 kb) [file 12974_2018_1356_MOESM1_ESM.pdf]

# Additional Figure 2

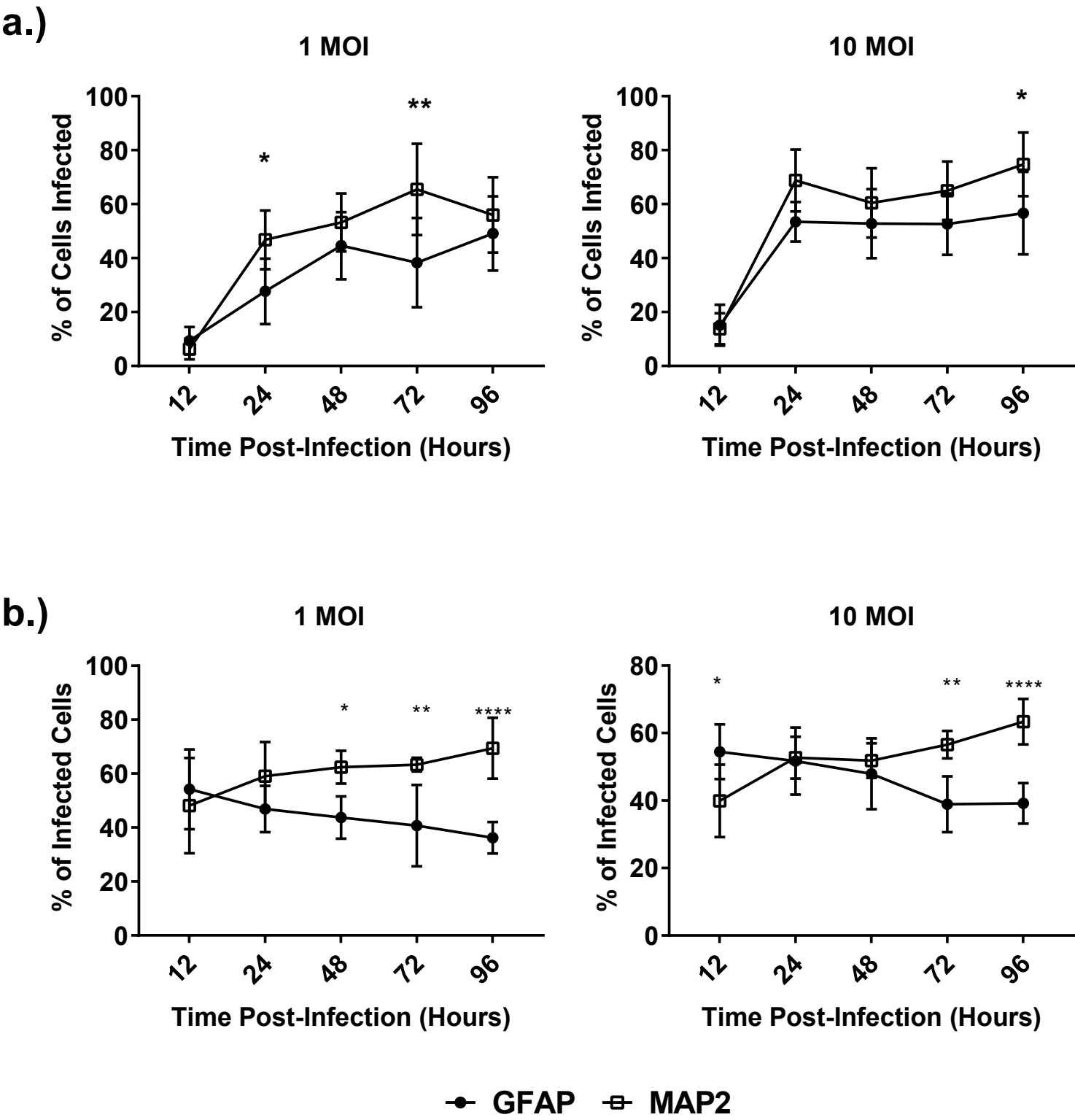

Supplement: Supplementary file 2 — Figure S2. Neurons and astrocytes are both targets of LACV infection across various MOIs. Neuron/astrocyte co-cultures were either mock infected or infected with 0.1 or 10 MOI of LACV. Cells were formalin fixed and stained for MAP2 or GFAP, LACV antigen and with DAPI counterstain. (a) Percentages of neurons and astrocytes infected with LACV were calculated (b) Percentages of infected cells positive for GFAP and MAP2 were calculated. *P < 0.05, **P < 0.01 (PDF 38 kb) [file 12974_2018_1356_MOESM2_ESM.pdf]

# Additional Figure 3

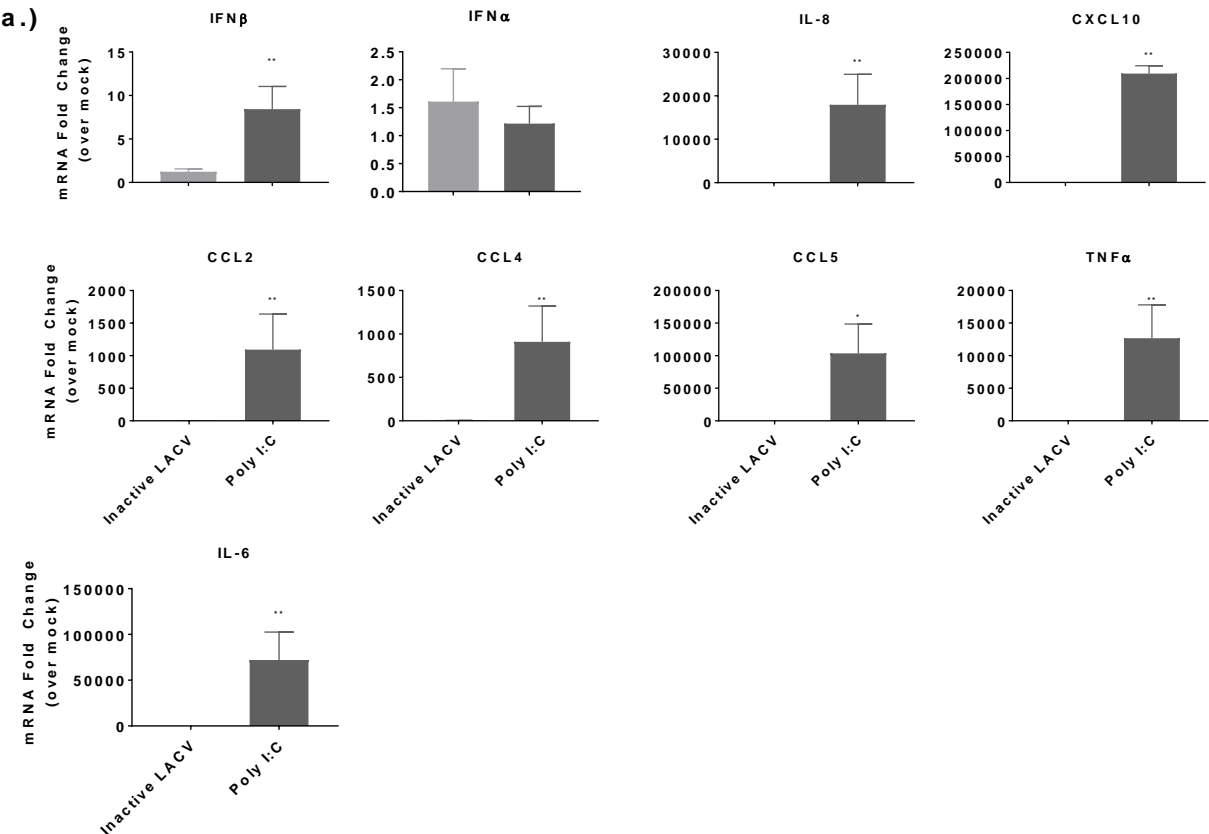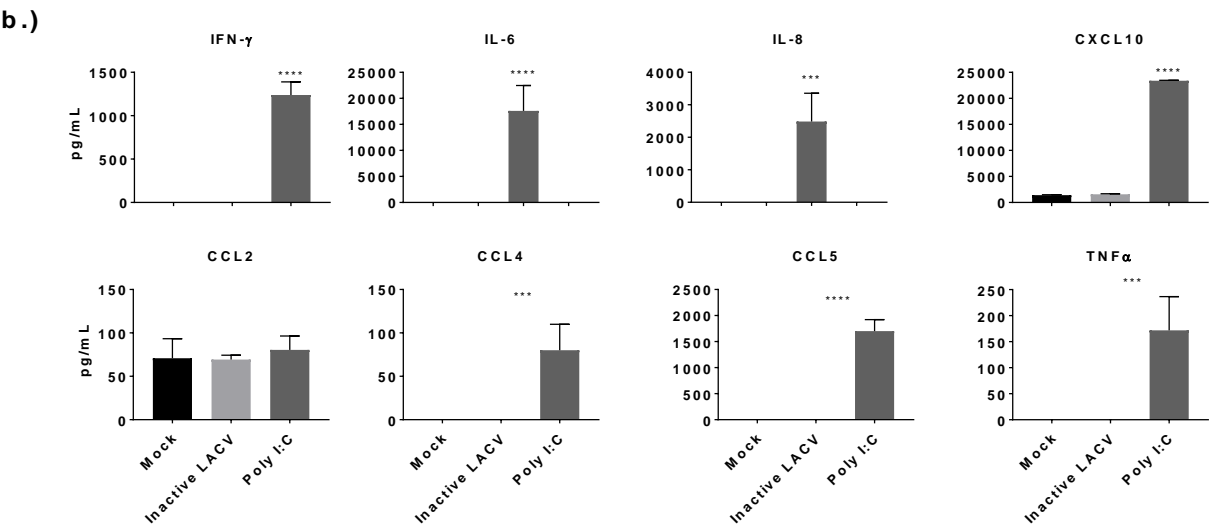

Supplement: Supplementary file 3 — Figure S3. Innate immune responses to inflammatory stimuli in neuron/astrocyte co-cultures. RT-PCR and BioPlex assays were performed to determine the cytokine and chemokine responses of neuron/astrocyte co-cultures. (a) Co-cultures were treated with either mock, Poly I:C, or heat inactivated LACV and at 48 HPI assessed for changes in gene expression for selected cytokines/chemokines via qRT-PCR. Values are reported as fold change relative to mock treatment normalized to 18S RNA. (b) Supernatant was collected and assayed for changes in selected cytokine/chemokine secretion via BioPlex assay. * P < 0.5, **P < 0.01, ***P < 0.001, ****P < 0.0001. (PDF 207 kb) [file 12974_2018_1356_MOESM3_ESM.pdf]

# Additional Figure 4

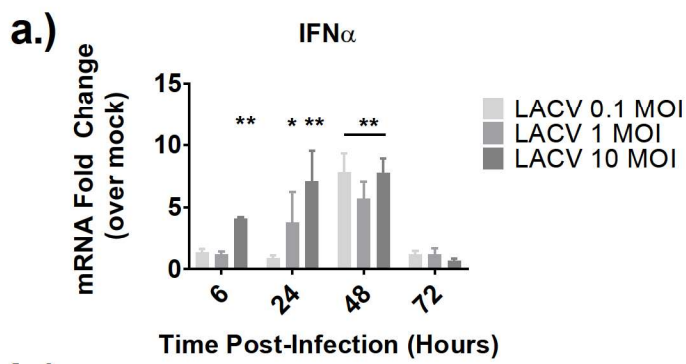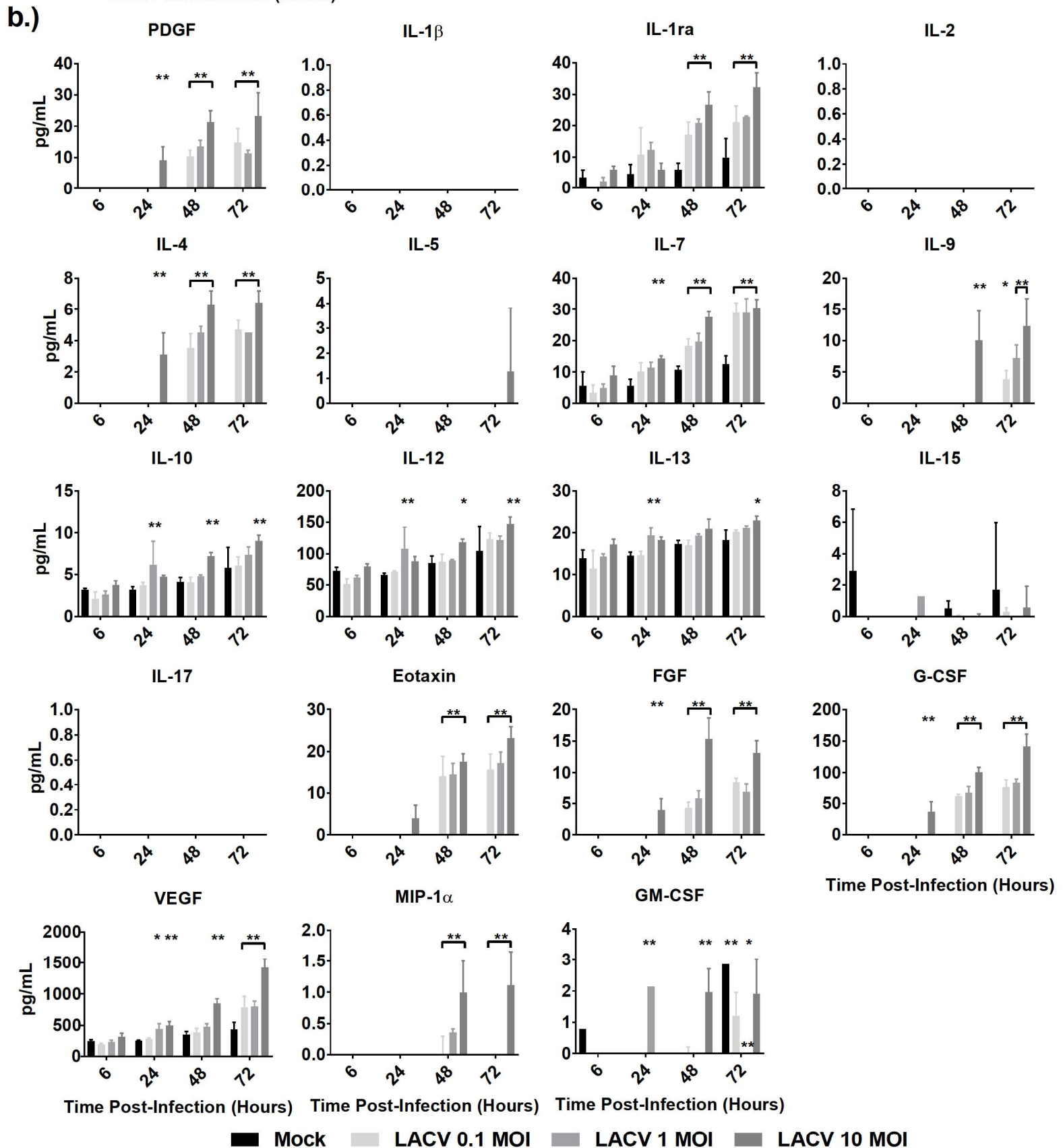

Supplement: Supplementary file 4 — Figure S4. Full cytokine and chemokine responses of neuron/astrocyte co-cultures to LACV infection. Cells were either infected with 0.1, 1, or 10 MOI of LACV. (a) Cells were lysed and RNA was collected and assessed for changes in selected cytokine/chemokine expression via qRT-PCR. Values are reported as fold change relative to mock treatment normalized to 18S. (b) Supernatant was collected and assayed for changes in selected cytokine/chemokine secretion via BioPlex assay *P < 0.5, **P < 0.01. (PDF 461 kb) [file 12974_2018_1356_MOESM4_ESM.pdf]
